# Inducible IL-7 Hyperexpression Influences Lymphocyte Homeostasis and Function and Increases Allograft Rejection

**DOI:** 10.3389/fimmu.2019.00742

**Published:** 2019-04-10

**Authors:** Maria Schreiber, Marc Weigelt, Anne Karasinsky, Konstantinos Anastassiadis, Sonja Schallenberg, Cathleen Petzold, Ezio Bonifacio, Karsten Kretschmer, Angela Hommel

**Affiliations:** ^1^Preclinical Approaches to Stem Cell Therapy/Diabetes, DFG-Center for Regenerative Therapies Dresden Cluster of Excellence, Center for Molecular and Cellular Bioengineering, Technische Universität Dresden, Dresden, Germany; ^2^Molecular and Cellular Immunology/Immune Regulation, DFG-Center for Regenerative Therapies Dresden Cluster of Excellence, Center for Molecular and Cellular Bioengineering, Technische Universität Dresden, Dresden, Germany; ^3^TU Dresden Faculty of Medicine, Paul Langerhans Institute Dresden, University Clinic Carl Gustav Carus, Helmholtz Centre Munich, Dresden, Germany; ^4^Stem Cell Engineering, BIOTEC, Technische Universität Dresden, Dresden, Germany

**Keywords:** Interleukin-7, allograft rejection, islet transplantation, Treg, immunosuppression

## Abstract

The IL-7/IL-7R pathway is essential for lymphocyte development and disturbances in the pathway can lead to immune deficiency or T cell mediated destruction. Here, the effect of transient hyperexpression of IL-7 was investigated on immune regulation and allograft rejection under immunosuppression. An experimental *in vivo* immunosuppressive mouse model of IL-7 hyperexpression was developed using transgenic mice (C57BL/6 background) carrying a tetracycline inducible IL-7 expression cassette, which allowed the temporally controlled induction of IL-7 hyperexpression by Dexamethasone and Doxycycline treatment. Upon induction of IL-7, the B220^+^ c-kit^+^ Pro/Pre-B I compartment in the bone marrow increased as compared to control mice in a serum IL-7 concentration-correlated manner. IL-7 hyperexpression also preferentially increased the population size of memory CD8^+^ T cells in secondary lymphoid organs, and reduced the proportion of CD4^+^Foxp3^+^ T regulatory cells. Of relevance to disease, conventional CD4^+^ T cells from an IL-7-rich milieu escaped T regulatory cell-mediated suppression *in vitro* and in a model of autoimmune diabetes *in vivo*. These findings were validated using an IL-7/anti-IL7 complex treatment mouse model to create an IL-7 rich environment. To study the effect of IL-7 on islet graft survival in a mismatched allograft model, BALB/c mice were rendered diabetic by streptozotocin und transplanted with IL-7-inducible or control islets from C57BL/6 mice. As expected, Dexamethasone and Doxycycline treatment prolonged graft median survival as compared to the untreated control group in this transplantation mouse model. However, upon induction of local IL-7 hyperexpression in the transplanted islets, graft survival time was decreased and this was accompanied by an increased CD4^+^ and CD8^+^ T cell infiltration in the islets. Altogether, the findings show that transient elevations of IL-7 can impair immune regulation and lead to graft loss also under immune suppression.

## Introduction

The IL-7/IL-7 receptor (IL-7R) pathway is indispensable for B and T cell development. IL-7 deficiency results in a severe block in B cell development at the transition from Pro-B to Pre-B cells in mice (1), and studies suggest that IL-7 is also critical for human B cell development (2). IL-7 is also an essential modulator of T cell homeostasis. In the steady state, the immune system relies on low concentrations of IL-7 to regulate T cell homeostasis and preserve T cell repertoire diversity (3, 4). During lymphopenia, an IL-7-rich environment provides a milieu for the proliferative expansion of T cells (3, 4).

IL-7-associated homeostatic expansion is also linked to inflammatory diseases, including graft-vs.-host-disease (5), rheumatoid arthritis (6), and multiple sclerosis (7). In murine models of autoimmune diabetes, IL-7 accelerates disease onset (8) and interference with IL-7 signaling prevents or even reverses disease (9–11). An increased concentration of IL-7 and homeostatic expansion of T cells, including autoreactive T cells, is observed in patients with Type-1-Diabetes (T1D) who receive immunosuppression as part of transplantation therapy (12). An increased IL-7 concentration also abrogates the ability of human FOXP3^+^ regulatory T (Treg) cells to suppress autoreactive effector T cell activation *in vitro* (13). IL-7 is, therefore, suggested to play a pivotal role in the development and recurrence of autoimmunity and graft failure.

A number of pathologies associated with increased IL-7 are associated with the concomitant treatment with immunosuppression, in particular after immune-depletion. Although animal models of increased IL-7 action exist (14–19), none of these includes hyper-IL-7 concentrations in an immunosuppressed environment. We, therefore, sought to develop such an *in vivo* mouse model and have used it to study IL-7 driven immune deviations under immunosuppressive conditions. In our model, IL-7 expression can be systemically induced at high levels resulting in bioactive IL-7 to drive population expansion. These findings with the model were validated using IL-7/anti-IL-7 mAb immune complexes, and altogether demonstrate that transient increases in IL-7 can impair immune regulation and decrease allograft survival.

## Materials and Methods

### Generation of Transgenic Mice

The eukaryotic expression vector ins-Hyg-tet-on-IL-7 was engineered for inducible IL-7 expression (Figure S1A). Transgenic mice were generated by pronuclear injection of the ins-Hyg-tet-on-IL-7 construct. Transgenic founder mice (tet-on-IL-7) were identified among offspring by genotyping using genomic PCR. Stable transgene integration was verified by breeding founder animals to C57BL/6 wild-type mice and subsequent genomic PCR-based genotyping of the offspring. To obtain mice with temporally controlled IL-7 hyperexpression, the C57BL/6.tet-on-IL-7 mouse line was crossed with C57BL/6.irtTA-GBD mice (20), giving rise to double transgenic C57BL/6.tet-on-IL-7-irtTA-GBD mice (dTG) as well as genotype control mice lacking either the tet-on-IL-7 or irtTA-GBD transgene (Ctrl) or both transgenes (WT). Additionally, C57BL/6.tet-on-IL-7-irtTA-GBD mice were crossed with C57BL/6.Foxp3^RFP/GFP^ mice (21), which are characterized by the double-transgenic expression of GFP (as a fusion protein with Cre recombinase) from a Foxp3-BAC [BAC.Foxp3^Cre−GFP^, (22)] and of RFP from an IRES downstream of the Foxp3 coding region [Foxp3^IRES−RFP^, (23)], to generate C57BL/6.tet-on-IL-7-irtTA-GBD × Foxp3^RFP/GFP^ mice. All mice were housed under specific pathogen-free conditions. All animal experiments were performed as approved by the Landesdirektion Dresden (24-9168.24-1/2012-7; DD24-5131/207/4; DD24.1-5131/354/90; DD24-5131/367/23; DD24.1-5131/394/45).

### Induction of an IL-7-Rich Environment *in vivo*

Male and female mice (dTG or Ctrl) were injected i.p. with 2 mg doxycycline (Dox, Sigma-Aldrich Chemie GmbH, Taufkirchen, Germany) and 0.5 mg dexamethasone (Dex, Sigma-Aldrich Chemie GmbH) per 25 g body weight on five consecutive days. Serum IL-7 concentration was measured before and after Dex/Dox administration, using the Mouse IL-7 Quantikine ELISA Kit according to the manufacturer's protocol (R&D Systems, Minneapolis, MN, USA).

To transiently induce high levels of IL-7 in mice without an inducible IL-7 transgene, male and female C57BL/6.Foxp3^RFP/GFP^ mice and NOD.Rag1^−/−^ recipients were i.p. injected with IL-7/anti- IL-7 mAb immune complexes (IL-7/M25 mice). For this purpose, 15 μg recombinant human IL-7 (rhIL-7, carrier-free, R&D Systems) were incubated with 1.5 μg anti-IL-7 mAb (clone M25, BioXCell, West Lebanon, NH, USA). After 30 min at 37°C, complexes were diluted with PBS to obtain 200 μL of IL-7 complex solution per mouse.

### mRNA Expression Analysis

Primary (bone marrow, BM; thymus) and secondary (spleen, SPL; lymph nodes, LNs) lymphoid organs were used for total RNA extraction, employing the RNeasy Mini Kit and DNase I digestion (Qiagen, Hilden, Germany), and cDNA was synthesized according to the manufacturer's recommendations (SuperScript II reverse transcriptase, Invitrogen, Thermo Fisher Scientific, Schwerte, Germany). The SYBR Premix exTaq kit (Takara Bio, USA) and a Mastercycler ep realplex thermal cycler (Eppendorf, Hamburg, Germany) were used to analyze cDNA in triplicates. Thefollowingprimers were used:HPRT, 5′-GTCAACGGGGGACATAAAAG-3′ and 5′-AGGGCATATCCAACAACAAAC-3′; beta-actin, 5′-TGGAATCCTGTGGCATCCATGAAA-3′ and 5′-TAAAACGCAGCTCAGTAACAGTCC-3′; IL-7, 5′-TCCCGCAGACCATGTTCCATGTTTC-3′ and 5′-TTCAACTTGCGAGCAGCACGA-3′.

### Flow Cytometry and Cell Sorting

Single cell suspensions of SPL, mesenteric LNs (meLNs), and a pool of subcutaneous LNs (scLNs: *Lnn. mandibularis, Lnn. cervicales superficiales, Lnn. axillares et cubiti, Lnn. inguinales superficiales*, and *Lnn. subiliaci*) were prepared using Hank's buffer [1 × HBSS, 5% (v/v) FCS, 10 mM HEPES; all Invitrogen] and 70 μm cell strainers (Becton Dickinson, San Diego, CA, USA). BM cells were harvested from femurs and tibias by flushing mechanically dissociated bones or cavities of intact bones with Hank's buffer, followed by filtration through 70 μm cell strainers. Single cell suspensions from SPL and BM were additionally subjected to red blood cell lysis (erythrocyte lysis buffer EL, Qiagen). mAbs to B220 (RA3-6B2), CD4 (RM4-5, GK1.5), CD8 (53–6.7), CD11b/Mac-1 (M1/70), CD11c (HL3), CD19 (1D3), CD21/CD35 (8D9), CD23 (B3B4), CD25 (PC61, 7D4), CD44 (IM7), CD45.1 (A20), CD45.2 (104), CD62L (MEL-14), CD117/c-kit (2B8), CD127 (SB/199, A7R34), Gr-1 (RB6-8C5), IgD (11–26c), IgM (II/41), NK1.1 (PK136), Sca-1 (D7), Ter119 (TER-119), as well as Pacific Blue, PE-Cy7 and PerCP-conjugated streptavidin were purchased from eBioscience (Frankfurt, Germany) or Becton Dickinson. Where indicated, CD4^+^, CD25^+^ or CD19^+^ cells were enriched from single cell suspensions using biotinylated mAbs directed against CD4, CD25 or CD19, streptavidin-conjugated microbeads, and the autoMACS or MultiMACS magnetic separation system (Miltenyi Biotec, Bergisch-Gladbach, Germany). For intracellular staining of Ki67 (SolA15; eBioscience), the Foxp3/Transcription Factor Staining Buffer Set (eBioscience) was used according to the manufacturer's protocol. Samples were analyzed on a FACSCalibur, FACS Aria III or sorted using a FACS Aria II and III (Becton Dickinson). Data were analyzed using FlowJo software (FlowJo LLC Ashland, OR, USA).

### B Cell Differentiation *in vitro*

For differentiation into surface IgM-expressing (sIgM^+^) cells, 3 × 10^4^ FACS-purified BM-derived B220^+^c-kit^+^CD19^+^ Pro/Pre-B I cells were seeded in 96-well round-bottom plates (Greiner bio-one GmbH, Frickenhausen, Germany) and cultured at 37°C and 5% CO_2_ in 200 μl Iscove's Modified Dulbecco's Media (IMDM) supplemented with 10% (v/v) FCS, 1 mM sodium pyruvate, 1 mM HEPES, 2 mM Glutamax, 100 U/ml Penicillin-Streptomycin, 0.1 mg/ml Gentamycine, 0.1 mM nonessential amino acids, and 0.55 mM β-mercaptoethanol (all Invitrogen) for up to 4 days, in the absence of added IL-7. Surface IgM expression was analyzed using flow cytometry.

### T Cell Culture

T cells were cultured in 96-well round-bottom plates (Greiner bio-one GmbH) at 37°C and 5% CO_2_ in 200 μl RPMI 1680 medium supplemented with 1 mM sodium pyruvate, 1 mM HEPES, 2 mM Glutamax, 100 U/ml Penicillin-Streptomycin, 0.1 mg/ml Gentamycine, 0.1 mM non-essential amino acids, 0.55 mM β-mercaptoethanol and 10% (v/v) FCS (all reagents Invitrogen). For *in vitro* suppression, CD4^+^CD62L^high^CD25^−^ T responder cells (Tresp) and CD4^+^CD25^+^Foxp3^RFP+^ Treg cells were FACS-isolated from peripheral lymphoid tissues. 5 × 10^4^ eFluor670-labeled (5 μM; eBioscience) Tresp cells were cultured in triplicate wells per condition and sample for 72 h with 2.5 × 10^5^ irradiated (30 Gy) T cell-depleted splenocytes and soluble anti-CD3ε mAb (1 μg/mL, 145-2C11; Becton Dickinson), either alone or with varying numbers of Treg cells as indicated.

### Adoptive Transfer Model of Autoimmune Diabetes

Autoimmune diabetes was induced in recipient mice by adoptive transfer of CD4^+^ T cells with transgenic expression of a diabetogenic T cell receptor. Conventional BDC2.5^+^ T cells with a naïve surface marker phenotype (CD4^+^BDC2.5^+^CD62L^high^CD25^−^) were isolated from pooled LNs and SPL of NOD.BDC2.5 mice by enrichment for CD4^+^ cells using MACS technology followed by FACS. 5 × 10^5^ diabetogenic cells were injected i.v. into NOD.Rag1^−/−^ mice. The *in vivo* suppressive capacity of Foxp3^+^BDC2.5^+^ Treg cells was assessed by co-injecting 1 × 10^5^ CD4^+^BCD2.5^+^CD25^+^Foxp3^RFP+^ cells that had been FACS-purified from pooled LNs and SPL of NOD.BDC2.5 × Foxp3^RFP/GFP^ mice. Blood glucose concentration of NOD.Rag1^−/−^ recipient mice were monitored for up to 30 days or until diabetes manifestation (blood glucose levels above 300 mg/dl on two consecutive measurements).

### Pancreatic Islet Isolation

Islets were isolated (24, 25) from the pancreas of 8-week-old dTG or littermate Ctrl donor mice by collagenase digestion (0.7 mg/ml) (Sigma-Aldrich Chemie GmbH) and discontinuous Ficoll density gradient. Islets were washed with RPMI-1640 medium supplemented with 1% (v/v) L-glutamine (Lonza Group, Basel, Switzerland), 1% (v/v) Penicillin-Streptomycin (Sigma-Aldrich Chemie GmbH), 5.5 mmol/l glucose (Sigma-Aldrich Chemie GmbH) and 5% (v/v) FCS (Gibco, Invitrogen, Paisley, UK); islets were then left to rest free-floating for 18 to 24 h at 37°C and 5% CO_2_, prior to transplantation.

### Culture of Pancreatic Islets

To determine the IL-7 release, islets of donor mice of C57BL/6.tet-on-IL-7-irtTA-GBD founders were freshly isolated. One Hundred islets were hand-picked underneath a microscope into open 1.5 ml Eppendorf tubes containing 200 μl RPMI-1640 medium. Islets were cultured in the absence or presence of Dex/Dox for 48 h with at 37°C and 5% CO_2_. The supernatant was harvested and stored at −80°C. IL-7 protein was detected in supernatants using a commercial mouse IL-7 ELISA kit (R&D Systems) according to the manufacturer's protocol.

### Allograft Model for Islet Transplantation

Eight-week-old BALB/c recipient mice (Charles River, Suelzfeld, Germany) were injected with streptozotocin (STZ, 225 mg/kg) (Sigma-Aldrich Chemie GmbH) to induce diabetes 5 to 7 days prior to islet transplantation. Mice that had a non-fasting blood glucose concentration above 400 mg/dl on two consecutive days were transplanted with 600 islets (dTG or littermate Ctrl) under the kidney capsule. Transplantation was performed using a 1001 TPLT Hamilton syringe (CS-Chromatographie Service, Langerwehe, Germany). Blood glucose was measured at 12 h intervals in the first 3 days post-transplantation. Normoglycemia was defined as non-fasting blood glucose levels < 200 mg/dl on at least two consecutive days. At day 7, normoglycemic transplanted recipients were i.p. injected with Dex/Dox for five consecutive days to transiently induce transgenic IL-7 expression in transplanted tet-on-IL-7-irtTA-GBD transgenic islets. Graft rejection was diagnosed when blood glucose increased to >200 mg/dl.

### Immunohistochemistry

Islet-bearing kidney cryosections (5 μm) were fixed in 4% formalin and stained for C-peptide using polyclonal rabbit anti-C-peptide (Cell Signaling, Boston, MA, USA), followed by Alexa Fluor 488-labeled polyclonal goat anti-rabbit IgG (Invitrogen) antibodies. Subsequently, detection of CD4 or CD8 was carried out using rat anti-CD4 (clone RM4-5) or rat anti-CD8 (clone 53-6·7) mAbs (Becton Dickinson), followed by staining with Alexa Fluor 568-labeled polyclonal goat anti-rat IgG secondary antibodies (Invitrogen). Nuclei were visualized by 4′-6-Diamidino-2-phenylindole (DAPI) staining. Slides were mounted with Vectashield (Vector Laboratories, Burlingame, CA, USA), using standard staining protocols. All images were acquired with a Leica SP5 upright Laser Scanning confocal microscope.

### Statistical Analysis

For statistical analysis, Prism 6.07 software (GraphPad Software, San Diego, CA, USA) was used. For statistical analysis of two groups, student's *t*-test was applied. For comparison of three or more groups, two-way ANOVA in combination with Bonferroni's multiple comparison post-test was used. For survival analysis, Log-rank (Mantel-Cox) test was used.

## Results

### Generation of Mice With Temporally Controlled IL-7 Hyperexpression

**Figure 1:**
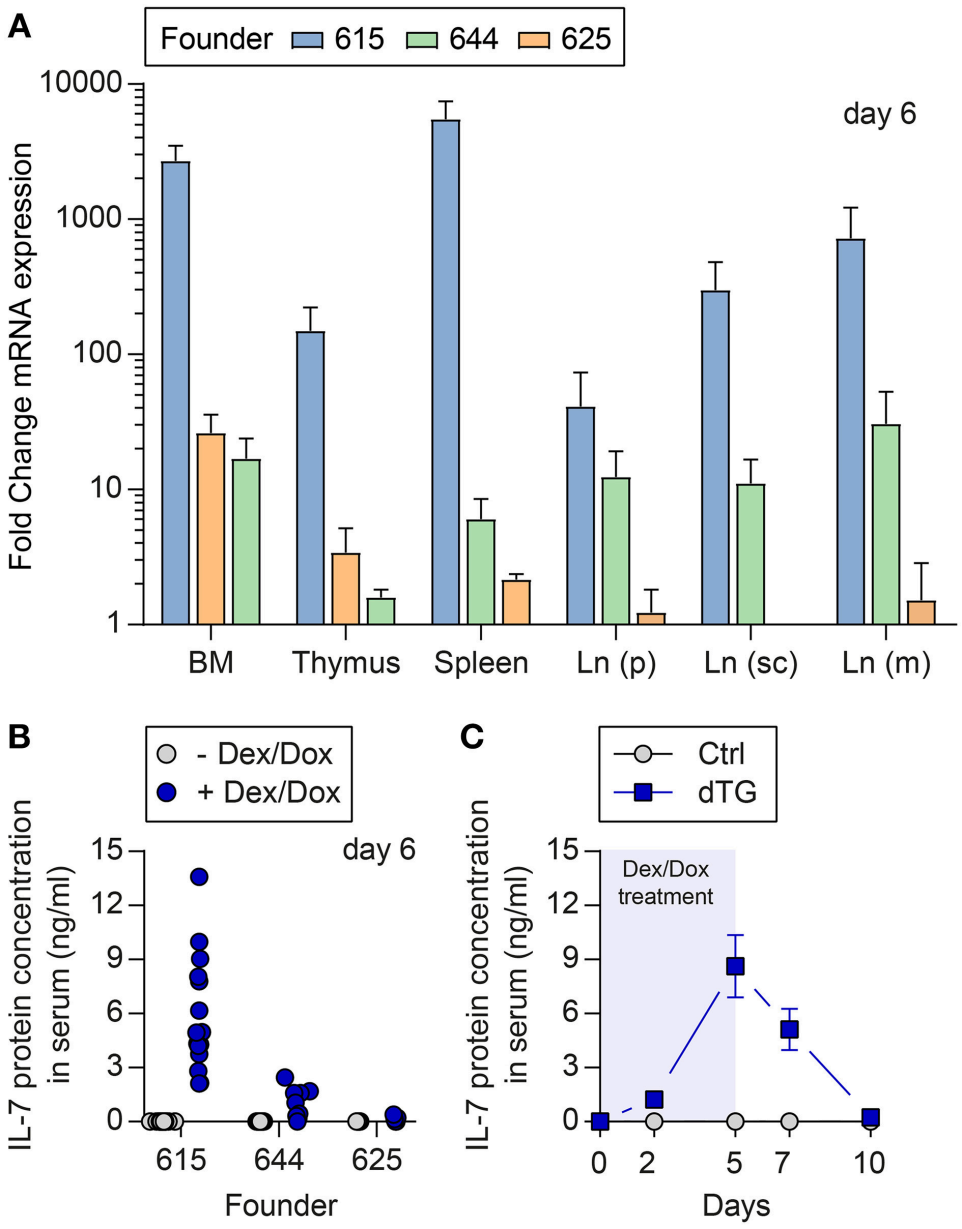
Temporally controlled induction of transgenic IL-7 expression *in vivo*. Dex/Dox-mediated induction (5 day treatment) of transgenic IL-7 expression in the three founder lines (615, 644, 625). **(A)** Induced *il7* mRNA expression was quantified by quantitative RT-PCR employing whole tissues harvested at day 5 from 8-week-old dTG mice (*n* = 5–6) of the indicated founder lines (blue bars: 615; green: 644; orange: 625). Shown are fold change values of dTG mice relative to Ctrl mice lacking the irtTA-GBD transgene (*n* = 3–6). **(B)** Serum was collected before (gray circles) and after (blue circles, day 5) Dex/Dox treatment. **(C)** Serum of dTG (blue squares) and Ctrl (gray circles) mice of the high expression founder 615 was collected at day 0, 2, 5, 7, and 10 after initiation of Dex/Dox administration and used for IL-7 protein quantification.

Three lines (founder number: 615, 625, and 644) had stable ins-Hyg-tet-on-IL-7 construct integration after breeding with C57BL/6 wild-type mice (Figure S1B). The C57BL/6.tet-on-IL-7 founder lines were crossed to C57BL/6.irtTA-GBD mice, giving rise to C57BL/6.tet-on-IL-7-irtTA-GBD mice (dTG) as well as genotype control mice (Ctrl) lacking either the tet-on-IL-7 or the irtTA-GBD transgene. Following Dex/Dox treatment of dTG and Ctrl mice for five consecutive days, all three lines exhibited induced expression (Figure S1C) of *il7* mRNA in multiple lymphoid organs (Figure 1A) and accumulation of IL-7 protein in serum (Figure 1B). The highest expression of mRNA and protein was observed in founder line 615 (Figures 1A,B). In time course studies (Figure 1C), serum IL-7 concentrations in founder line 615 peaked on the last day of Dex/Dox treatment (day 5) and declined to concentrations similar to baseline levels within 5 days after discontinuation of Dex/Dox treatment.

### IL-7 Expression Levels Correlate With the Population Size of BM Pro/Pre-B I Cells

**Figure 2:**
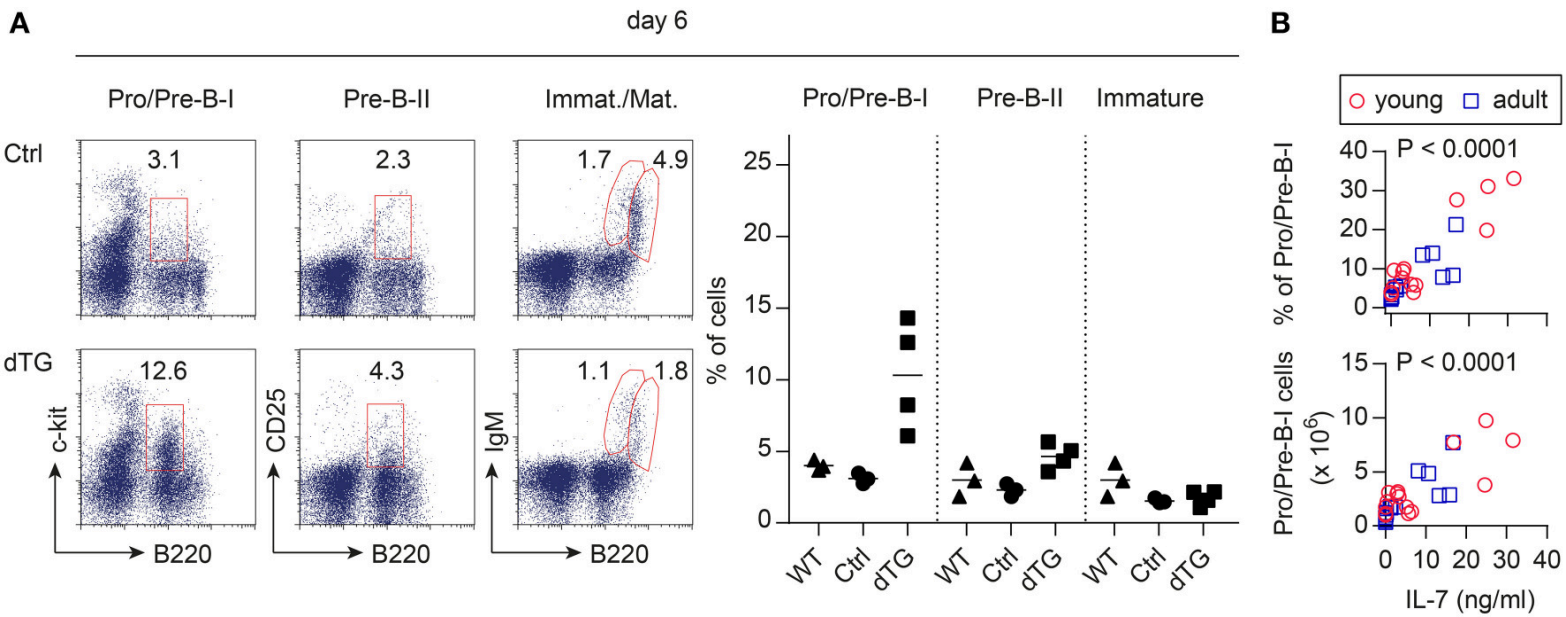
Induced IL-7 expression enhances B lymphopoietic activity in the BM. IL-7 hyperexpression was induced by Dex/Dox for 5 days and BM was analyzed by flow cytometry at day 6. **(A)** Representative flow cytometry (left) of B220^+^c-kit^+^ Pro/Pre-B I, B220^+^CD25^+^ Pre-B II, immature B220^low^sIgM^+^, and mature/recirculating B220^high^sIgM^+^ B cells of adult Ctrl (mice expressing only the irtTA-GBD transgene) and dTG mice. Graphs (right) depict composite percentages of respective cell population out of total lymphocytes in replicate Ctrl, dTG, and untreated WT mice that were siblings of the Ctrl and dTG mice. Symbols and horizontal lines indicate individual mice and mean values, respectively. **(B)** Percentage (top panel) and cell numbers (lower panel) of Pro/Pre-B I cells in the BM as depicted in **(A)** from young (red circles) and adult (blue squares) dTG mice of all three founder lines (#615, 644, 625) plotted against serum concentrations of IL-7 protein for individual mice to provide a dose range. *P*-values were calculated from the Pearson correlation coefficient.

To assess the bioactivity of induced IL-7 hyperexpression *in vivo*, B lymphopoietic activity was analyzed (Figure 2). BM from young (4-week-old) and adult (16-week-old) mice was harvested at different time points following the initiation of IL-7 by Dex/Dox treatment. On day 6, the percentage of the B220^+^c-kit^+^ Pro/Pre-B I cell compartment [nomenclature according to (26)] increased by approximately 3-fold upon IL-7 induction in the dTG line 615 as compared to Ctrl mice (Figure 2A, left; Ctrl: 3.1 ± 0.3%, dTG: 10.3 ± 3.3%; *P* = 0.024). No increase in earlier B cell developmental stages was observed (Figure S2A). Overall, Pro/Pre-B I cell frequencies and counts differed between the three dTG lines (#615, 625, and 644; Figure 2B) but correlated with serum concentrations of IL-7 protein (*r* = 0.83; *P* < 0.0001). All subsequent experiments were performed with the dTG line #615.

On day 10, the frequencies of Pro/Pre-B I cells in the BM of the dTG line 615 were similar to those observed in Ctrl mice, while the compartment size of B220^+^CD25^+^ Pre-B II cells and immature B220^+^IgM^low^ B cells increased in dTG mice (Figure S2B, *P* = 0.0012; *P* = 0.0005), suggesting developmental progression toward subsequent developmental stages after initial proliferative expansion of Pro/Pre-B I cells (27, 28). No increase in mature CD19^+^ splenocytes was observed (Figure S2C). FACS-isolated BM-derived B220^+^CD19^+^c-kit^+^ Pro/Pre-B I cells of dTG mice cultured in the absence of added IL-7efficiently differentiated into sIgM^+^ cells, albeit with delayed upregulation of sIgM, as compared to their counterparts from Ctrl mice (Figure S2D; *P* < 0.0001).

We conclude that the induced hyperexpression of IL-7 in dTG mice appears suitable to modulate IL-7-dependent biological processes *in vivo*.

### Induced IL-7 Hyperexpression *in vivo* Drives Expansion of CD8^+^ T Cells With a Memory Phenotype in Young Mice

**Figure 3:**
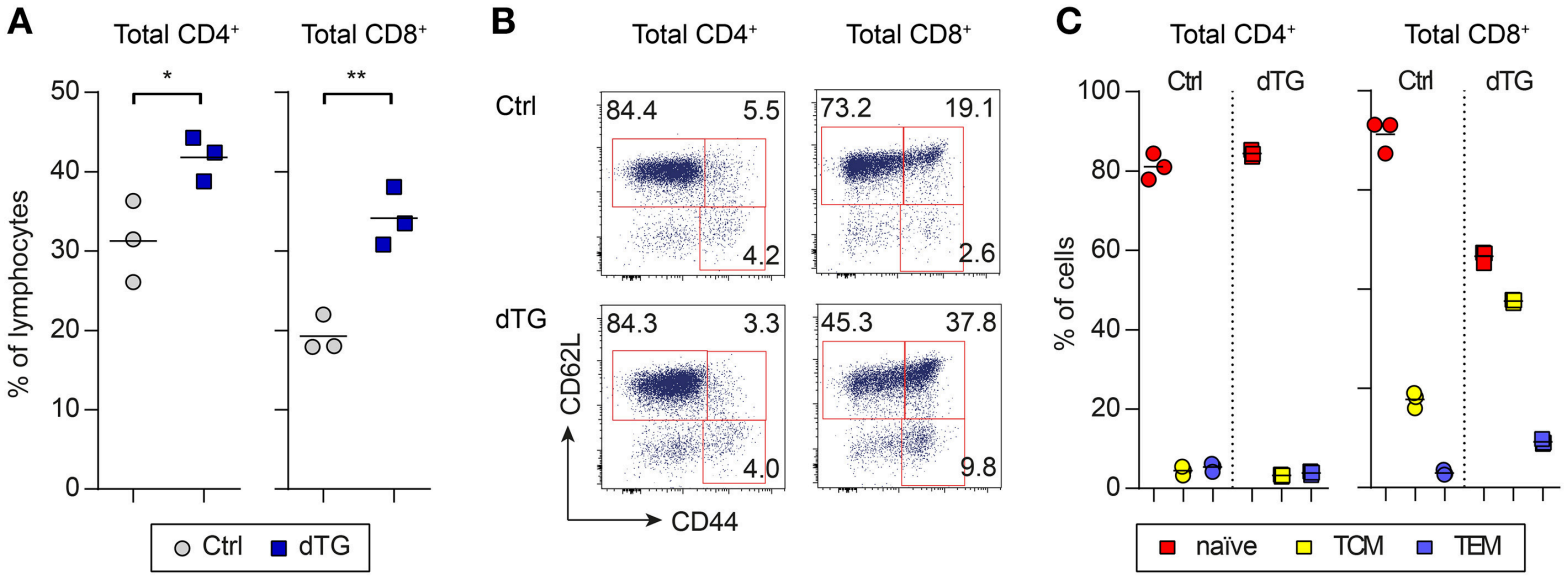
IL-7 hyperexpression preferentially expands CD8^+^ T cell populations with a memory phenotype. IL-7 hyperexpression was induced in young (4-week-old) and adult (16-week-old) mice (both Ctrl and dTG) by 5 days Dex/Dox treatment and peripheral lymphoid tissues were analyzed at day 6. **(A)** Flow cytometry of CD4^+^ and CD8^+^ T cells from scLNs of young mice. Graphs depict the frequency of the indicated T cell compartment out of total CD4^+^ or CD8^+^ T cells. **(B)** Representative flow cytometry of subpopulations among total CD4^+^ and CD8^+^ T cells (naïve, CD62L^+^CD44^−^; TCM, CD62L^+^CD44^+^, central memory; TEM, CD62L^−^CD44^+^, effector memory). Numbers in dot plots indicate percentages of cells in the respective gates. **(C)** Relative distribution of CD4^+^ (left) and CD8^+^ (right) T cells with a naïve, TCM, or TEM phenotype, as gated in **(B)**. Symbols and horizontal lines in **(A)** and **(C)** indicate individual mice and mean values, respectively. Results obtained from adult (16-week-old) Ctrl and dTG mice are depicted in Figure S3. Absolute numbers are shown in Figure S4. *P < 0.05; **P < 0.01.

IL-7 is essential for the survival and homeostasis of T cells. Consistent with this, scLN of young dTG mice at day 6 after initiation of Dex/Dox treatment had increased proportions (Figure 3A) as well as absolute numbers (Figure S4) of total CD4^+^ T cells (Ctrl: 31.3 ± 5.1%; dTG: 41.8 ± 2.8%; *P* = 0.035) and CD8^+^ T cells (Ctrl: 19.3 ± 2.3%; dTG: 34.1 ± 3.7%; *P* = 0.0042), as compared to Ctrl mice. Among CD4^+^ T cells, the relative distribution of cells with a naïve (CD62L^+^CD44^−^), central memory (TCM, CD62L^+^CD44^+^) and effector memory (TEM, CD62L^−^CD44^+^) phenotype remained unaffected by IL-7 hyperexpression (Figures 3B,C, left; *P* > 0.05). In contrast, the CD8^+^ T cell population in scLN from Dex/Dox-treated dTG mice showed a shift from cells with a naïve phenotype (Ctrl: 71.2 ± 3.3%; dTG: 46.6 ± 1.2%; *P* = 0.0003) to CD8^+^ T cells with a TCM (Ctrl: 17.8 ± 1.6%; dTG: 37.6 ± 0.4%; *P* < 0.0001) and a TEM (Ctrl: 3.0 ± 0.5%; dTG: 9.2 ± 0.5%; *P* < 0.0001) phenotype as compared to Dex/Dox-treated Ctrl mice (Figures 3B,C, right). A similar shift for CD8^+^ T cells was also observed in meLNs and SPL of young mice (Figures S3A, S4). Increased proportions of CD4^+^ and CD8^+^ T cells were observed in scLN of Dex/Dox-treated dTG adult mice, but without changes in the relative proportions of phenotypes (Figure S3B). IL-7 induced proliferation of CD4^+^ and CD8^+^ T cells was confirmed by Ki67 staining in mice that were treated with rhIL-7/anti-IL-7 mAb (clone: M25) immunocomplex (IL-7/M25) that mimics an IL-7-rich environment without additional Dex/Dox-mediated immunosuppression (Figure S5).

### Differential Impact of Increased IL-7 *in vivo* on Foxp3^+^ Treg Cell-Mediated Suppression of T Effector Cell Activity *in vitro*

**Figure 4:**
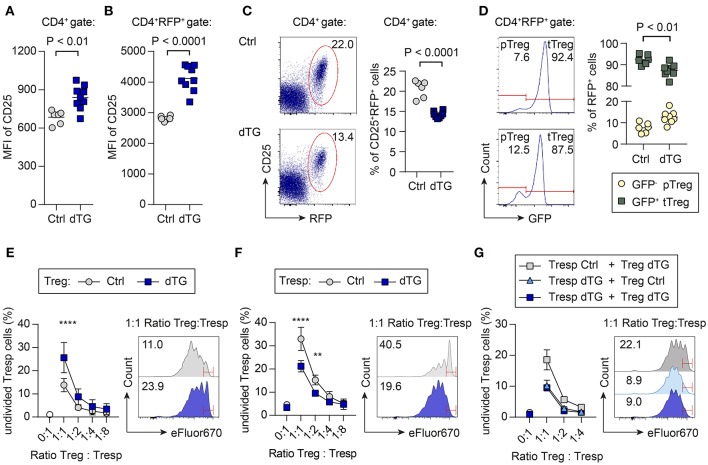
Differential impact of increased IL-7 *in vivo* on CD25 expression, p/tTreg cell abundance, and Foxp3^+^ Treg cell-mediated suppression *in vitro*. To track viable populations of Foxp3^+^ Treg cells, we employed C57BL/6.tet-on-IL-7-irtTA-GBD × Foxp3^RFP/GFP^ mice, in which all Foxp3^+^ Treg cells express RFP and developmental sublineages can be distinguished based on differential GFP expression (tTreg, RFP^+^GFP^+^; pTreg, RFP^+^GFP^−^) (21). IL-7 hyperexpression was induced by 5 day Dex/Dox treatment, and scLNs were isolated on day 6. **(A–D)** Impact of IL-7 hyperexpression *in vivo* on Foxp3^+^ Treg cells. CD25 expression levels among **(A)** total CD4^+^ T cells and **(B)** CD4^+^-gated total Foxp3.RFP^+^ Treg cells from Dex/Dox-treated Ctrl (for separate results in Ctrl irtTA-GBD and Ctrl tet-on-IL-7 single TG mice see Figure S6) and dTG mice, as revealed by the flow cytometric assessment of median fluorescent intensity (MFI). **(C)** Representative flow cytometry of Foxp3.RFP^+^ expression among gated CD4^+^ T cells. **(D)** Relative contribution of GFP^−^ pTreg and GFP^+^ tTreg cells among total CD4^+^RFP^+^ Treg cells. Symbols and horizontal lines in **(A–D)** indicate individual mice and mean values, respectively. Numbers in dot plots and histograms indicate the percentage of cells within the respective gates. **(E–G)** Impact of transgenic IL-7 hyperexpression *in vivo* on Treg cell-mediated suppression of conventional CD4^+^ T cell activation *in vitro*. Naïve CD4^+^CD62L^high^CD25^−^ T responder (Tresp) cells and CD4^+^CD25^+^RFP^+^ Treg cells were FACS-purified from peripheral lymphoid tissues of the indicated mice (Ctrl, dTG; ± Dex/Dox). eFluor670-labeled Tresp cells were then cultured with irradiated T cell-depleted splenocytes, in the absence or presence of T cell stimulatory anti-CD3ε mAbs, either alone or co-cultured with Treg cells at varying Tresp:Treg ratios. Graphs depict the composite percentage of undivided Tresp cells under different culture conditions and Tresp:Treg cell ratios (triplicate wells per condition and sample), as indicated. Histograms show representative flow cytometry of eFluor670 dilution by Tresp cell proliferation at a Tresp:Treg ratio of 1:1; numbers in histograms indicate the percentage of undivided Tresp cells within the indicated gates. **(E)** Tresp cells were isolated from untreated mice, Treg cells were derived from Dex/Dox-treated Ctrl or dTG mice, as indicated. Symbols in the graph show mean values ± SD of two independent experiments: Ctrl: pool of 2–3 mice, total of 10 mice; dTG: pool of 1–2 mice, total of 7 mice. **(F)** Tresp cells were isolated from Dex/Dox-treated Ctrl and dTG mice, and Treg cells from untreated mice, as indicated. Symbols in the graph show mean values ± SD of triplicate wells of one representative of two independent experiments: Ctrl: pool of 2 mice, total of 4 mice; dTG: total of 2 mice. **(G)** Both Tresp and Treg cells were isolated from Dex/Dox-treated mice (either Ctrl or dTG), as indicated. Shown are mean and range of triplicate wells from one experiment. Ctrl: pool of 3 mice, dTG: pool of 2 mice. For statistical analysis, two-way ANOVA with Bonferroni's multiple comparisons test was applied. **P ≤ 0.01; ****P ≤ 0.0001.

Dex/Dox treatment of dTG mice increased CD25 expression levels on total CD4^+^ T cells in scLNs (Figure 4A; MFI Ctrl: 684 ± 53; MFI dTG: 839 ± 93; *P* = 0.0027). To directly track Treg cells based on Foxp3 expression, C57BL/6.tet-on-IL-7-irtTA-GBD founder line 615 was crossed to C57BL/6.Foxp3^RFP/GFP^ mice, in which all Foxp3^+^ Treg cells express RFP (21, 29). In Dex/Dox treated C57BL/6.tet-on-IL-7-irtTA-GBD × Foxp3^RFP/GFP^ mice, CD25 expression on Foxp3^IRES−RFP+^ Treg cells was increased (Figure 4B; MFI Ctrl: 2817 ± 80; MFI dTG: 4124 ± 421; *P* < 0.0001), but the proportion of Foxp3^IRES−RFP+^ Treg cells among total CD4^+^ T cells was consistently decreased in peripheral lymphoid tissues (Figure 4C; Ctrl: 20.6 ± 2.1%; dTG: 14.2 ± 0.8%; *P* < 0.0001).

C57BL/6.Foxp3^RFP/GFP^ mice allow discrimination of Foxp3.RFP^+^ Treg cell sublineages of thymic (tTreg, RFP^+^GFP^+^) and peripheral (pTreg, RFP^+^GFP^−^) developmental origin based on differential GFP expression (21, 29). IL-7 hyperexpression moderately increased the contribution of Foxp3.RFP^+^GFP^−^ pTreg cells (Figure 4D; Ctrl: 7.6 ± 2.3%; dTG: 12.6 ± 3.0%; *P* = 0.0056) at the expense of Foxp3.RFP^+^GFP^+^ tTreg cells (Ctrl: 92.4 ± 2.3%; dTG: 87.4 ± 3.0%; *P* = 0.0056). We did not observe differences between irtTA-GBD and tet-on-IL-7 single TG control mice (Figure S6).

Similar changes in Treg frequency and p/tTreg ratio were observed in scLNs, meLNs and SPL of both young and adult mice (Figures S7A,B). These findings were confirmed using rhIL-7/anti-IL-7 mAb (clone: M25) immunocomplex (IL-7/M25) treatment in C57BL/6.Foxp3^RFP/GFP^ mice (Figure S7C), indicating that the modulation of Treg cell homeostasis by elevated IL-7 bioactivity *in vivo* is consistent and independent of an immunosuppressive environment.

We subsequently assessed the impact of transgenic IL-7 hyperexpression *in vivo* on Foxp3^+^ Treg cell-mediated suppression of conventional CD4^+^Foxp3^−^ T cell activation in standard co-cultures, employing varying ratios of Treg and T responder (Tresp) cells (Figures 4E–G). Treg cells from Dex/Dox-treated dTG mice (IL-7 exposed) suppressed the proliferation of CD4^+^ Tresp cells from untreated (i.e., no Dex/Dox) mice more efficiently than their counterparts isolated from Dex/Dox-treated Ctrl donors (Figure 4E, e.g., 1:1 ratio: Treg Ctrl: 13.8 ± 2.8% undivided Tresp cells; Treg dTG: 25.6 ± 6.5% undivided Tresp cells; *P* < 0.0001). Tresp cells from Dex/Dox-treated dTG mice were also more resistant to suppression by Treg cells from untreated mice *in vitro* as compared to Tresp cells from Dex/Dox-treated Ctrl mice (Figure 4F; e.g., 1:1 ratio: Tresp Ctrl: 33 ± 5% undivided Tresp cells; Tresp dTG: 21.2 ± 2.4% undivided Tresp cells; *P* < 0.0001). To determine whether increased Treg suppression or Tresp resistance was dominant, suppression assays were performed with Tresp and Treg cells isolated from Dex/Dox-treated dTG or Dex/Dox-treated Ctrl mice (Figure 4G). When both Tresp and Treg cells were isolated from dTG mice, the IL-7 effect of enhanced Tresp cell resistance to Treg cell-mediated suppression *in vitro* could not be overcome by the observed IL-7-mediated increase in Treg cell suppressor function (e.g., 1:1 ratio: 9.5 ± 0.5% undivided dTG Tresp cells, *P* < 0.0001).

These findings were validated by isolating Treg and Tresp cells from an IL-7-rich milieu without additional immunosuppression (i.e., from mice treated with IL-7/M25 immune complexes; Figure S7D), indicating that elevated IL-7 availability *in vivo* modulates Treg and Tresp cell function *in vitro*.

### Increased IL-7 Promotes Resistance to Treg Cell-Mediated Suppression of Diabetes Induction

**Figure 5:**
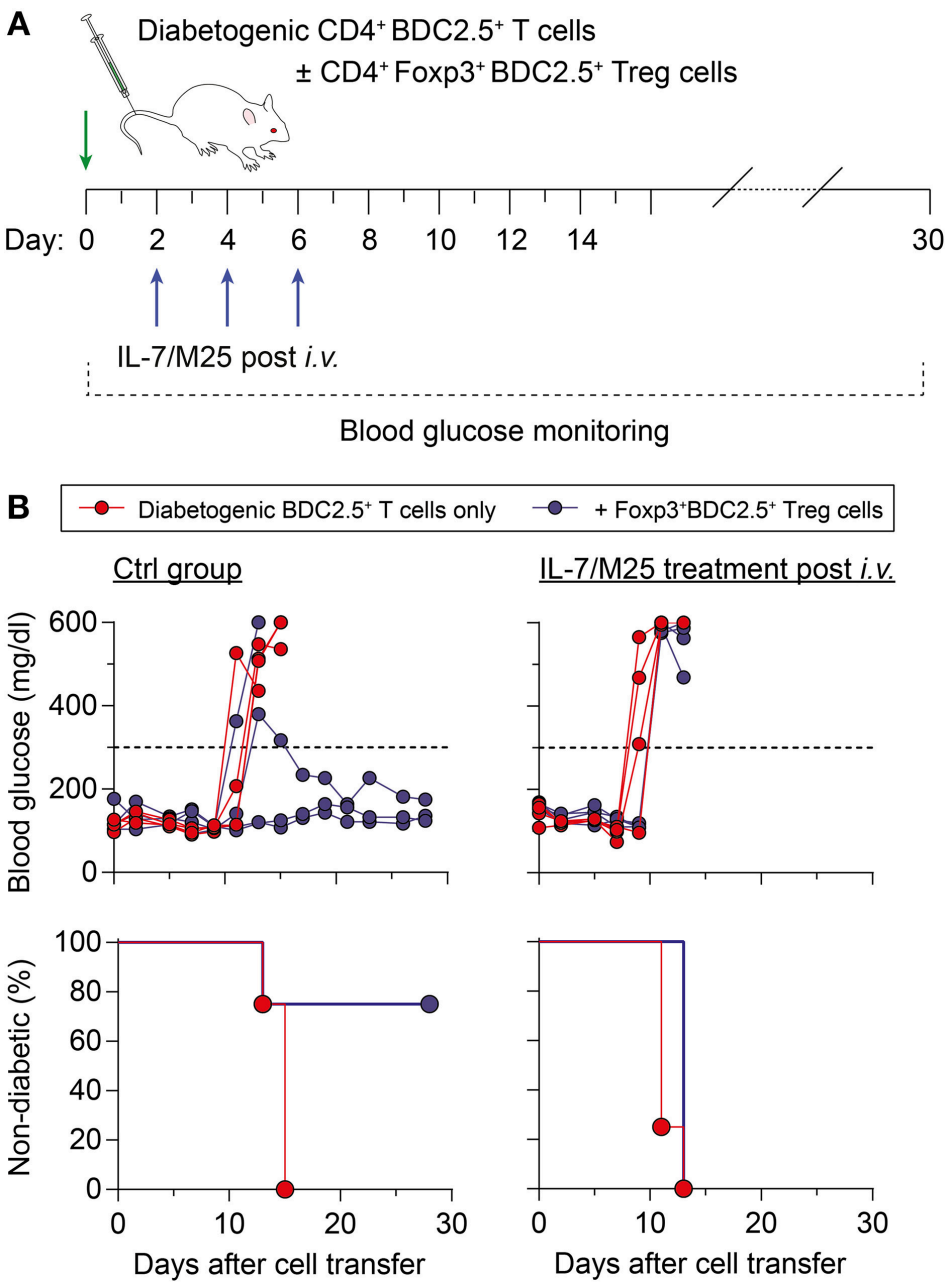
Increased IL-7 *in vivo* promotes resistance to Foxp3^+^ Treg cell-mediated immune regulation in an adoptive transfer model of autoimmune diabetes. **(A)** Schematic workflow of adoptive BDC2.5^+^ T cell transfer for diabetes induction. Diabetogenic CD4^+^BDC2.5^+^ T cells with a naïve CD25^−^CD62L^high^ phenotype were FACS-purified from pooled SPL and LNs of NOD.BDC2.5 mice, and 5 × 10^5^ cells were i.v. injected into NOD.Rag1^−/−^ recipients (day 0), either alone (*n* = 4) or co-transferred with CD4^+^CD25^+^Foxp3^RFP+^BDC2.5^+^ Treg cells (1 × 10^5^ cells/mouse; n = 4) isolated from NOD.BDC2.5 × Foxp3^RFP/GFP^ mice. Recipient mice were left untreated (Ctrl group) or received IL-7/M25 immune complexes by three i.v. injections (d2, d4, d6). Blood glucose was measured every other day for up to 30 days. **(B)** Blood glucose concentration (top) and diabetes incidence (bottom) of NOD.Rag1^−/−^ recipient mice that only received BDC2.5^+^ T cells (left) or were additionally treated with IL-7/M25 following adoptive T cell transfer (right). Mice were considered to have diabetes at blood glucose levels above 300 mg/dl [dashed lines in **(B)**, top] on at least two consecutive measurements.

Next, we determined the suppressor function of Treg cells in an IL-7-rich environment in a model of autoimmune diabetes, in which co-transfer of CD4^+^Foxp3^+^BDC2.5^+^ Treg cells suppress diabetogenic CD4^+^BDC2.5^+^ T effector cell-mediated destruction of pancreatic beta cells in NOD.Rag1^−/−^ recipients (30). For this purpose, recipient mice were either left untreated (Ctrl group) or received IL-7/M25 immune complexes following adoptive T cell transfer (post i.v. group) (Figure 5A). Injection of 5 × 10^5^ diabetogenic CD4^+^BDC2.5^+^ T cells with a naïve surface marker phenotype resulted in overt diabetes at day 14.5 ± 1.0 in recipient mice of the otherwise untreated Ctrl group (Figure 5B, left), while co-transfer of as few as 1 × 10^5^ BDC2.5^+^Foxp3^+^ Treg cells were sufficient to suppress diabetes development. IL-7/M25 treatment of recipient mice starting as early as day 2 after cell transfer shortened the lapse of time until diabetes development to day 11.5 ± 1.0 if only diabetogenic CD4^+^BDC2.5^+^ T cells were transferred (Figure 5B, right; *P* < 0.05). Interestingly, co-transfer of Foxp3^+^BDC2.5^+^ Treg cells did not prevent diabetes induction in recipient mice as observed for the control group. Here, overt diabetes was detected at day 13.0 ± 0.0 (*P* < 0.05). Hence, increasing IL-7 was able to increase the pathogenicity of antigen-specific T cells, enabling them to escape Treg cell-mediated suppression in an autoimmune diabetes model.

### Local IL-7 Hyperexpression in Transplanted Insulin-Producing Islets Is Sufficient to Promote Allograft Rejection

**Figure 6:**
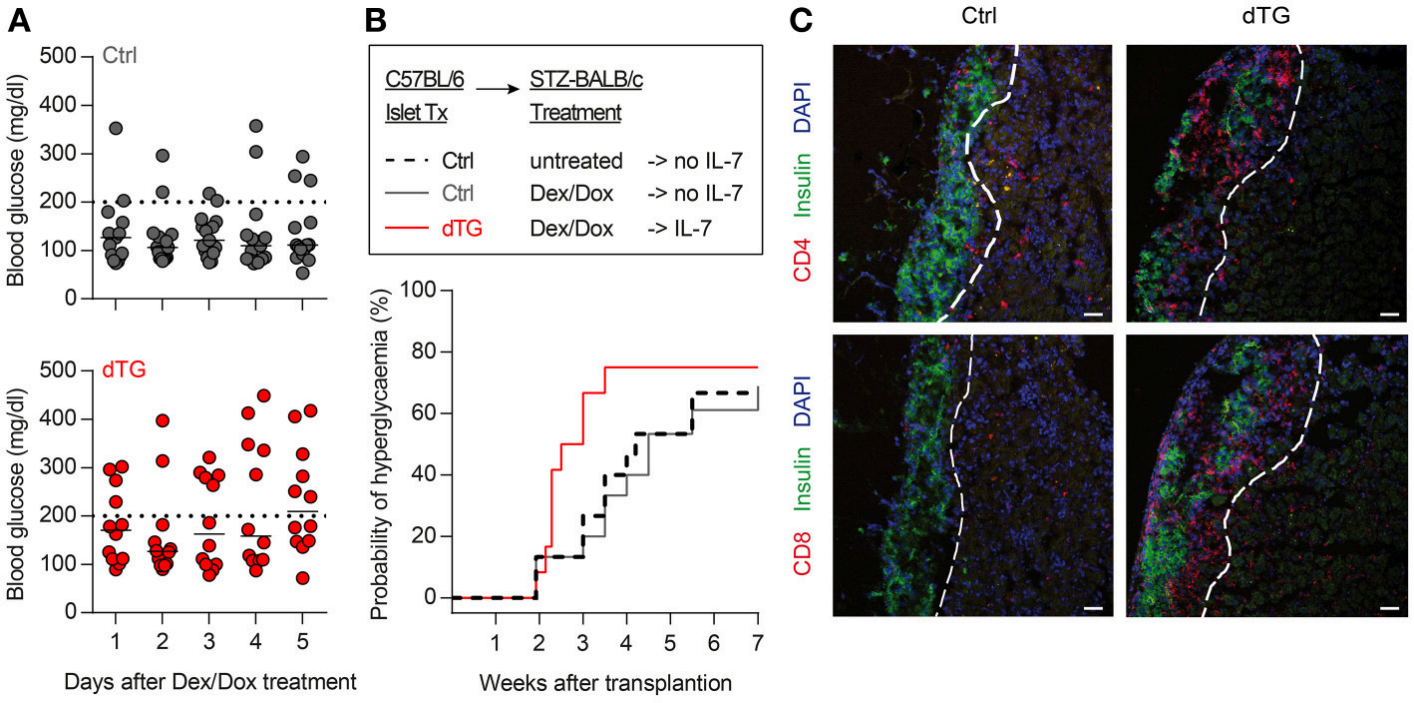
Local IL-7 hyperexpression in transplanted islets accelerates allograft rejection. **(A)** Median blood glucose concentrations after initiation of 5-day Dex/Dox treatment of BALB/c recipients (*n* = 11) transplanted with 600 allogenic islets from either Ctrl (upper) or dTG (lower) mice under the kidney capsule. **(B)** Kaplan–Meier analysis for allograft rejection-mediated hyperglycemia in C57BL/6 islets 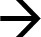 BALB/c recipients under different experimental conditions, as indicated: dashed black line: C57BL/6-Ctrl islets without Dex/Dox treatment (*n* = 15); solid dark gray line: Ctrl-islets and Dex/Dox treatment (total *n* = 15; thereof irtTA-GBD n = 10 and tet-on-IL-7 *n* = 5; for separate data in these groups, see Figures S8B, C); solid red line: dTG islets and Dex/Dox treatment (*n* = 12). Hyperglycemia was defined as blood glucose levels >200 mg/dl on consecutive days [horizontal dotted lines in **(A)**]. **(C)** Representative histology of C57BL/6 beta cells (C-peptide, green) of indicated transplanted islets under the kidney capsule of STZ-pre-treated BALB/c recipients, either in combination with infiltrating CD4^+^ (red, upper panels) or CD8^+^ (red, lower panels) T cells. Cell nuclei are visualized using DAPI (blue). Histological images are representative of three mice; scale bar: 100 μm. Broken white line = border between transplanted islets and kidney capsule.

C57BL/6.tet-on-IL-7-irtTA-GBD mice offer an opportunity to study the role of IL-7 in modulating immunity *in vivo* in various experimental settings with clinical relevance. Here, we used the model to assess the impact of IL-7 hyperexpression in the microenvironment of insulin-producing islet transplants in the setting of allograft rejection (C57BL/6 islets 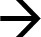 BALB/c recipients). Islets isolated from dTG mice released detectable amounts of IL-7 when cultured in the presence of Dex/Dox (Figure S8A). *In vivo*, BALB/c mice with STZ-induced diabetes were transplanted with C57BL/6 islets isolated from either dTG or Ctrl donor mice. Mice transplanted with dTG-islets and subsequently treated with Dex/Dox had increased blood glucose concentrations as compared with mice that had received Ctrl-islets (247 ± 102 mg/dl after IL-7 induction vs. 151 ± 75 mg/dl control islets; *P* < 0.05) (Figure 6A). Under immunosuppressive conditions (i.e., after Dex/Dox administration) the rejection of the transplanted islets defined as blood glucose levels > 200 mg/dl was more frequent in mice that received IL-7 releasing islets (63.6% [95% CI 37.6%, 81.1%] at day 20) than in recipients of islets from control mice (18.2% [95% CI 0.1%, 56.4%]; *P* < 0.05) (Figure 6B). Reduced graft survival was associated with increased infiltration of CD4^+^ (Figure 6C, upper) and CD8^+^ (Figure 6C, lower) T cells in transplanted dTG islets with transgenic IL-7 hyperexpression compared to Ctrl-islets. Thus, the selective induction of IL-7 hyperexpression in transplanted dTG islets was sufficient to accelerate allograft rejection, abrogating the immunosuppressive effect of Dex/Dox on graft survival. Foxp3 staining was not performed to determine whether this was due to a reduction in infiltrating Tregs.

## Discussion

Cell replacement therapy in T1D must consider strategies to control immune-mediated loss of graft tissue due to reoccurring autoimmunity and graft rejection. Using an immunosuppressive pre-clinical model with transiently inducible IL-7 hyperexpression, we verified inducibility and bioactivity of the transgenic IL-7 protein, demonstrated irregularities in immunoregulation following IL-7 hyperexpression, and showed that local islet IL-7 hyperexpression promoted CD4^+^ and CD8^+^ T cell infiltration leading to enhanced graft rejection.

The mouse model, which features both inducible on-off expression and immune-suppression, showed typical responses to increased IL-7 seen in other models and systems (14–19). Immunophenotyping of our dTG mice revealed a profound increase in Pro/Pre-B-I cells in the BM by IL-7 hyperexpression, while retaining their developmental potential. Constitutively or transiently elevated IL-7 expression without additional immunosuppression was also previously shown to increase this B cell progenitor cell population (14, 31, 32). In secondary lymphoid organs, CD8^+^ T cells with a memory surface phenotype were preferentially expanded in an IL-7-rich environment. A predominance of CD8^+^ to CD4^+^ T cells was also detected in mice with constitutive transgenic IL-7 expression under the control of the MHC class II promoter, which showed a CD44^+^ memory phenotype (17, 33), independent of IL-15 signaling (33). While we did not address effector functions of the expanded memory-like CD8^+^ T cells, others have previously shown that *in vivo* exposure to high levels of transgenically expressed IL-7 led to a higher proportion of IFN-γ-producing cells among the memory CD8^+^ T cells (33). This is also in line with observations from lymphopenia-derived memory T cells, which upregulated CD44 and elicited effector functions, including rapid IFN-γ secretion (34–36). In parallel, IL-7 hyperexpression in an immunosuppressive environment reduced the proportion of Treg cells among total CD4^+^ T cells, confirming previously published findings in IL-7/anti-IL-7 mAb immunocomplex treated mice (37). Hence, we predict that our model, which also include immunosuppression, recapitulates many of the previously reported effects of increased IL-7 activity on B and T lymphocytes. A limitation of our study was that we did not include control experiments in which the activity of transgene derived IL-7 was blocked by antibodies or other means and it, therefore, may be possible that some of the observed effects are not strictly due to IL-7. We also mainly used a mix of two single transgenic animals that had no excess IL-7. These single transgenic animals appeared to be similar in their response to Dex/Dox treatment, but large numbers of each of these were not tested.

Crossing of our inducible mouse model with the Foxp3^RFP/GFP^ reporter mouse model (21) provided further evidence that IL-7 promotes Foxp3^+^ Treg cell homeostasis by increasing the pTreg contribution to the overall Treg cell pool. In contrast to our own studies of human Treg cells in the presence of IL-7 *in vitro* (13), an *in vivo* exposure to an IL-7-rich environment improved Treg suppressive capacity *in vitro*. Whether this discrepancy is due to *in vitro* or *in vivo* IL-7 exposure or a due to differences between humans and mice needs to be further elucidated. However, this improved Treg suppressive function was outcompeted by the enhanced resistance of Tresp cells to Treg cell-mediated suppression in an IL-7 rich environment. Several studies have described the phenomenon of reduced Tresp cell sensitivity to the suppressive function of Treg cells in various autoimmune diseases, including T1D (38), systemic lupus erythematosus (39), rheumatoid arthritis (40), and juvenile idiopathic arthritis (41), which also have been shown to be associated with lymphopenia and elevated IL-7 levels (42). The mechanism of the observed Tresp cell resistance is unclear. The PI3K/AKT signaling pathway as a downstream target of IL-7/IL-7R signaling and SHP-1 are shown to affect Tresp resistance to Treg (43–45). Bockade of the STAT5-dependent co-inhibitory receptor LAG3 leads to enhanced homeostatic proliferation of adoptively transferred CD4^+^ T cells in a lymphopenic host, increased CD25 expression and Tresp cell resistance to Treg-mediated suppression *in vitro* and *in vivo* (46). Moreover, Vazquez-Mateo and colleagues showed that blockade of IL-7Rα increased LAG3 as well as PD-1 and Tim-3 expression on CD4^+^ T cells rendering them more susceptible for co-inhibitory signals (11). Further studies should address whether IL-7 hyperexpression in our model affects PI3K/Akt and SHP-1 pathways, as well as LAG3 and/or other co-inhibitory receptor expression on Tresp cells.

We previously showed that IL-7 levels are elevated post islet transplantation in humans leading to the proliferation of memory T cell clones despite immunosuppression (12). Using isolated islets from our dTG mice in a genetically mismatched alloimmune model (C57BL/6 islet transplantation in diabetic BALB/c mice), we showed that induction of locally restricted IL-7 hyperexpression leads to enhanced CD4^+^ and CD8^+^ T cell infiltration resulting in augmented graft rejection. These results indicate that IL-7 can directly contribute to allograft rejection. Age is of potential relevance for the magnitude of the effect. Our experiments showed stronger effects of IL-7 hyperexpression on T cells in 4-week-old mice than 16-week-old mice. The transplant experiments were performed on 8-week-old mice. Islet transplantation is generally performed in adult patients and an increase in IL-7 may not be of major consequence in adults. On the other hand, increases in IL-7 may be relevant to early autoimmunity in type 1 diabetes, which occurs in young children (47), and where T cells are highly sensitive to IL-7 (48).

In conclusion, we successfully established a pre-clinical model with inducible bioactive IL-7 hyperexpression. We showed that the induced IL-7 protein affects lymphocyte development, homeostasis and function at multiple levels in the model and can aggravate allograft rejection. This model can be used to pre-clinically test therapies in transplantation relevant settings.

## Ethics Statement

This study was carried out in accordance with the recommendations of Landesdirektion Dresden, Ethikkommission an der TU Dresden. The protocol was approved by the Ethikkommission an der TU Dresden.

## Author Contributions

MS, EB, AH, KA, and KK designed the study, contributed to the conduct of the study, the acquisition, analysis, and interpretation of data, and drafted, reviewed, and approved the manuscript. MW, AK, SS, and CP contributed to the acquisition, analysis and interpretation of data, and approved the manuscript. AH, EB, and KK are the guarantors of this work.

### Conflict of Interest Statement

The authors declare that the research was conducted in the absence of any commercial or financial relationships that could be construed as a potential conflict of interest.
