# Supplementary material for: Inducible IL-7 Hyperexpression Influences Lymphocyte Homeostasis and Function and Increases Allograft Rejection
Source: Front Immunol. 2019 Apr 10;10:742. doi: 10.3389/fimmu.2019.00742 (PMC6467976; doi:10.3389/fimmu.2019.00742)
Supplement: Supplementary file 1 [file Data_Sheet_1.pdf]

frontiers

Supplementary Material

Inducible IL-7 Hyperexpression Influences Lymphocyte Homeostasis and Function and Increases Allograft Rejection

Maria Schreiber[1,3], Marc Weigelt[1,2], Anne Karasinsky[1], Konstantinos Anastassiadis[4], Sonja Schallenberg[3], Cathleen Petzold[3], Ezio Bonifacio[1,2,*], Karsten Kretschmer[2,3,*,‡], Angela Hommel[1,2 ,*, ‡]

*** Correspondence:**
Prof. Ezio Bonifacio, PhD
ezio.bonifacio@tu-dresden.de

Prof. Karsten Kretschmer, PhD
karsten.kretschmer@tu-dresden.de

Dr. Angela Hommel
angela.hommel@tu-dresden.de

1 Supplementary Figures

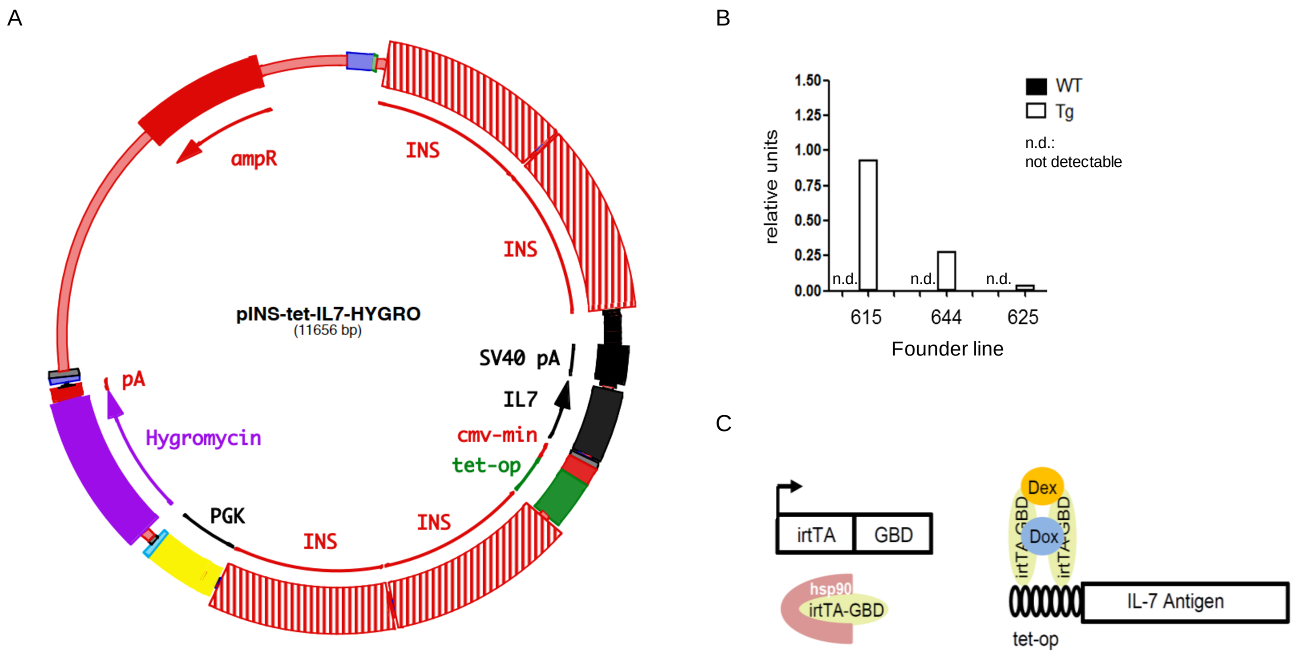

Figure S1. **Generation of the inducible mouse model of IL-7 hyperexpression. (A)** Plasmid chart. For inducible IL-7 expression, the eukaryotic expression vector ins-Hyg-tet-on-IL-7 was constructed using standard methods. In brief, cloned cDNA encoding the murine IL-7 was inserted into the pSPORT1-vector, and the CMV-Tet-Ins2-Hyg fragment was added 5' of the IL-7 cDNA. An INS-INS fragment was then inserted into the Sal I sites 3' of the IL-7 poly(A) signal sequence. **(B)** Quantitative PCR on genomic DNA to confirm IL-7 transgene integration and estimate copy number. Genomic integration of the ins-Hyg-tet-on-IL-7 expression construct was assessed by quantitative PCR on DNA harvested from 3-week-old wildtype (wt: black bars; n.d.: below detection limit) and transgenic (tg: white bars) mice. **(C)** Schematic illustration of the mechanism of IL-7 hyperexpression induction. Left: the activator complex irtTA-GBD (improved reverse tetracycline transactivator fused to a glucocorticoid binding domain) is constitutively expressed, but remains inactive in the cytoplasm due to its association with the heatshock protein 90 (HSP90). Right: upon binding of Dexamethasone (Dex) to the GBD domain, the activator complex dissociates from HSP90 and is able to translocate into the nucleus. Here, Doxycycline (Dox) enables the complex to bind to the tet-operator (tet-op) driving the expression cassette of the IL-7 transgene.

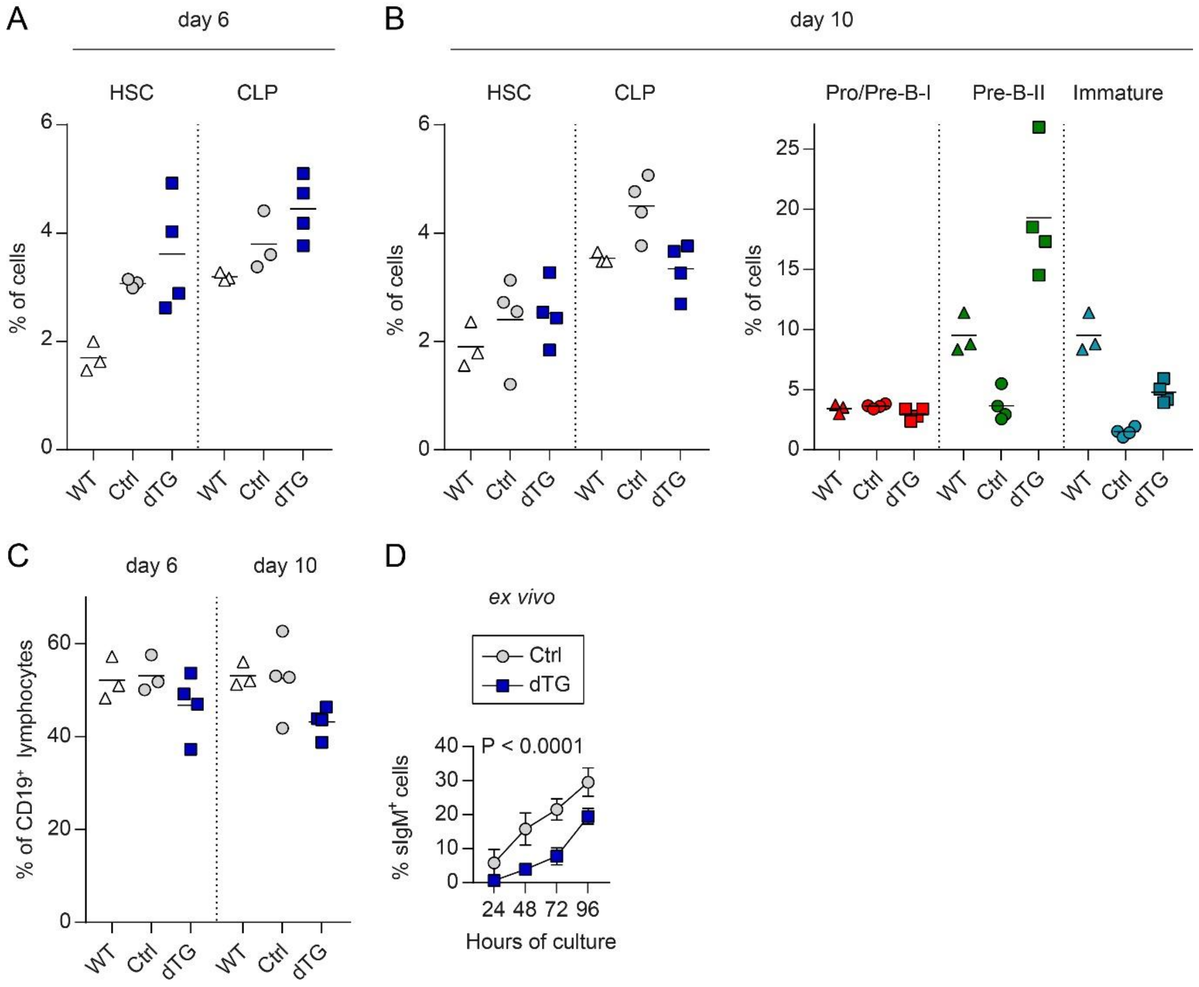

Figure S2. Induced IL-7 expression enhances B lymphopoietic activity in the BM. IL-7 hyperexpression was induced by Dex/Dox for 5 days in Ctrl (mice expressing only the irtTA-GBD transgene) and dTG mice and BM was analyzed by flow cytometry **(A)** at day 6 as shown in Figure 2. WT mice that are siblings of the Ctrl and dTG mice, were left untreated. Graphs depict composite percentages of HSC (hematopoietic stem cells: Sca-1^{high}, c-kit^{high}) and CLP (common lymphoid progenitor: Sca-1^{-}, c-kit^{med}) out of the lineage negative population ($CD4^{-}$ $CD8^{-}$ $CD19^{-}$ $CD11b^{-}$ $CD11c^{-}$ $Ter119^{-}$ $NK1.1^{-}$ Gr-1^{-}) in replicate WT, Ctrl and dTG mice. **(B)** Composite percentages of B cell developmental stages as described in (A) and Figure 2 at day 10 after initiation of Dex/Dox treatment. **(C)** Analysis of $CD19^{+}$ lymphocytes in SPL at day 6 and day 10. Symbols and horizontal lines indicate individual mice and mean values, respectively. **(D)** Differentiation capacity of Pro/Pre-B I cells from Dex/Dox treated Ctrl and dTG mice *in vitro*. BM-derived $B220^{+}$ c-$kit^{+}CD19^{+}$ Pro/Pre-B I cells were purified and cultured in the absence of added IL-7. Their sIgM expression status was assessed by flow cytometry. Symbols represent mean values of at least three biological replicates ± SD. Shown is a representative of two independent experiments. Two-way ANOVA.

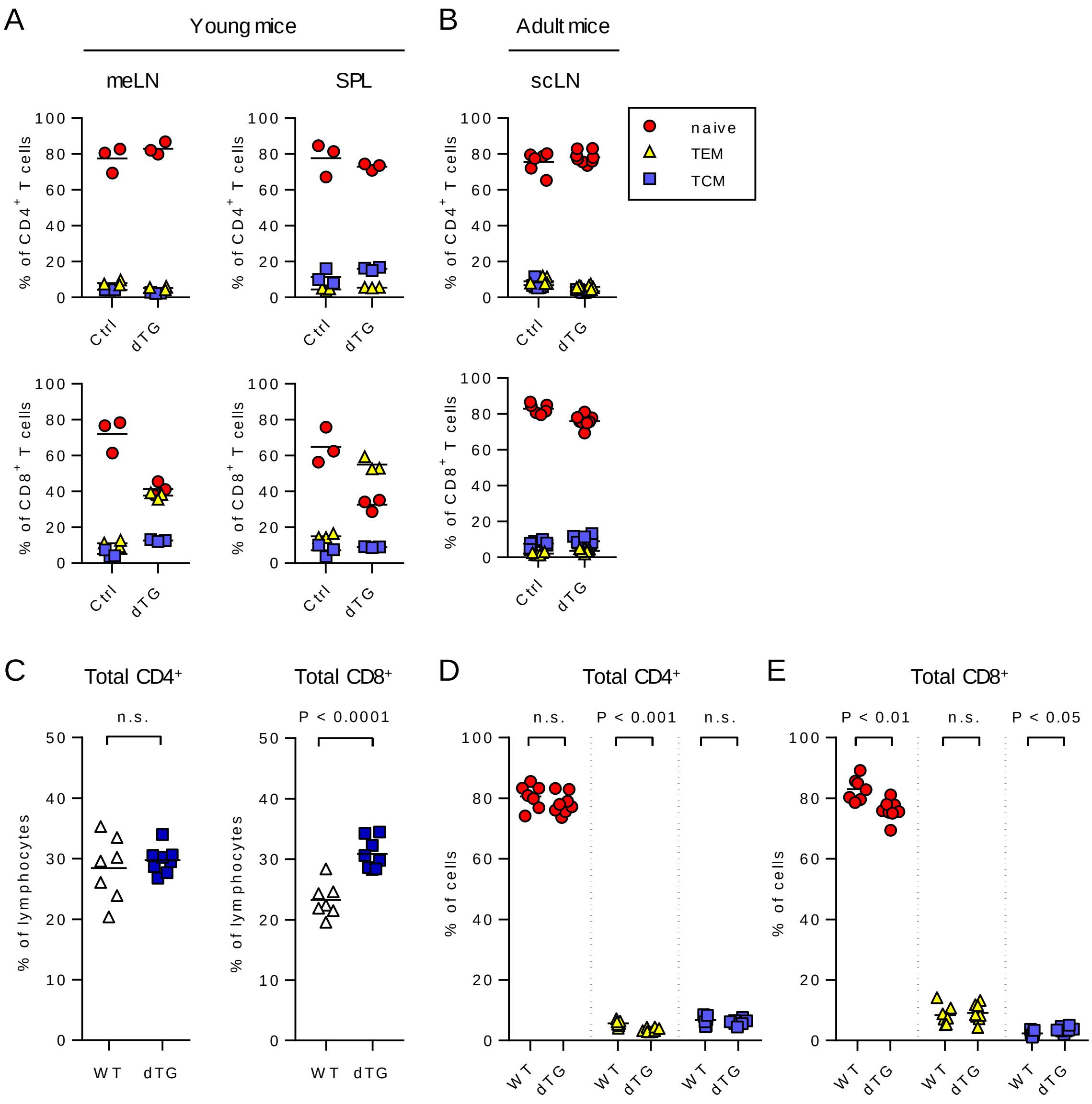

Figure S3. **IL-7 hyperexpression preferentially expands CD8$^+$ T cell populations with a memory phenotype**. IL-7 hyperexpression was induced in **(A)** young (4-week-old) mice and **(B)** adult (16-week-old) mice by 5-day Dex/Dox treatment and scLNs, meLNs and SPL were analyzed at day 6 by flow cytometry. Graphs depict the relative distribution of subpopulations of total CD4$^+$ (upper row) and CD8$^+$ (lower row) T cells in Ctrl and dTG mice. **(C, D, E)** Comparison CD4$^+$ and CD8$^+$ T cell populations in scLN of adult dTG mice (n = 8) to untreated WT mice (n = 7) that were siblings of the dTG mice. Red circles: naïve, CD62L$^+$CD44$^-$; yellow triangles: TCM, CD62L$^+$CD44$^+$, central memory; light blue squares: TEM, CD62L$^-$CD44$^+$, effector memory phenotype. Symbols and horizontal lines indicate individual mice and mean values, respectively.

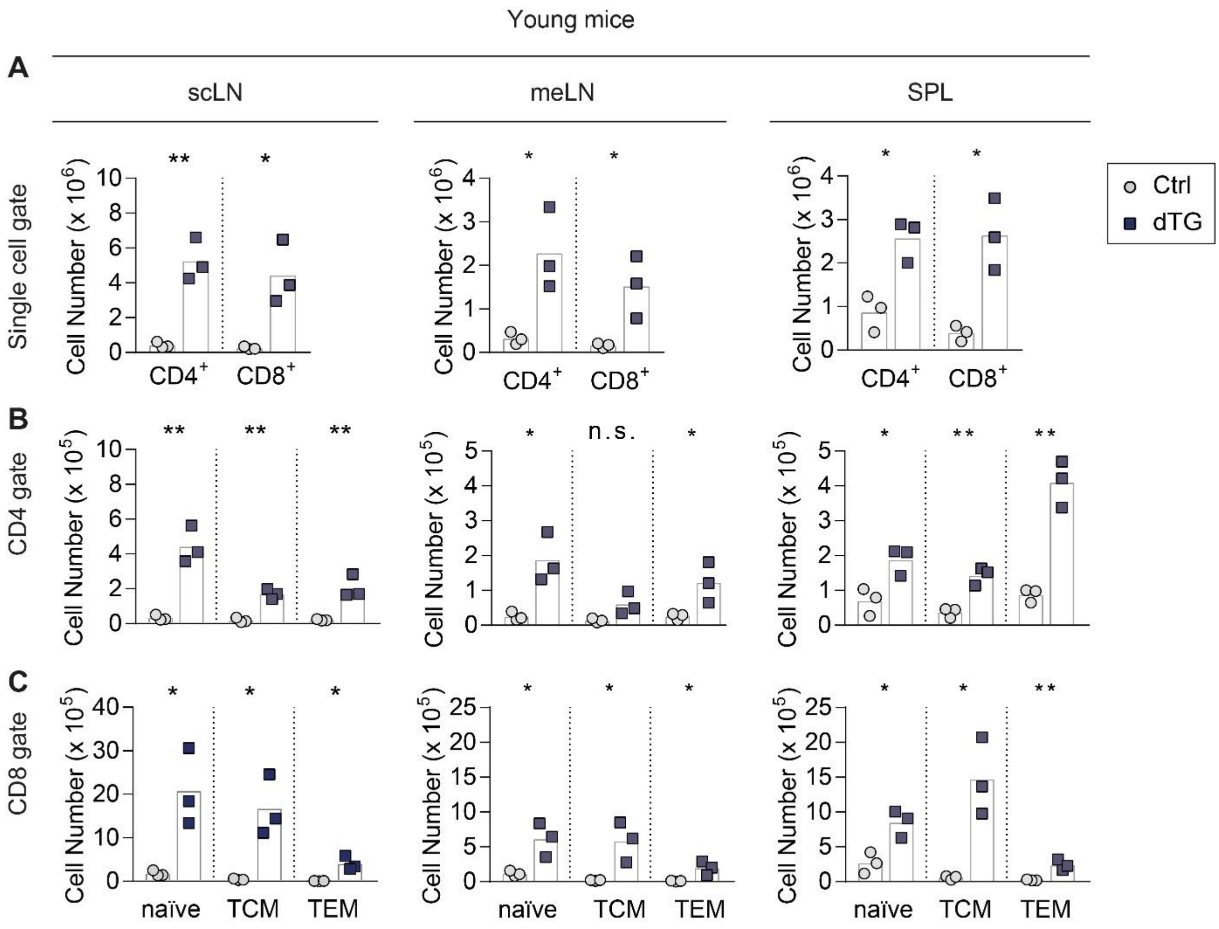

Figure S4. Absolute numbers for **(A)** total $CD4^+$ and $CD8^+$ T cell population as well as **(B)** $CD4^+$ and **(C)** $CD8^+$ subpopulations in scLN, meLN and SPL of young mice as shown Figure 3 and S3.

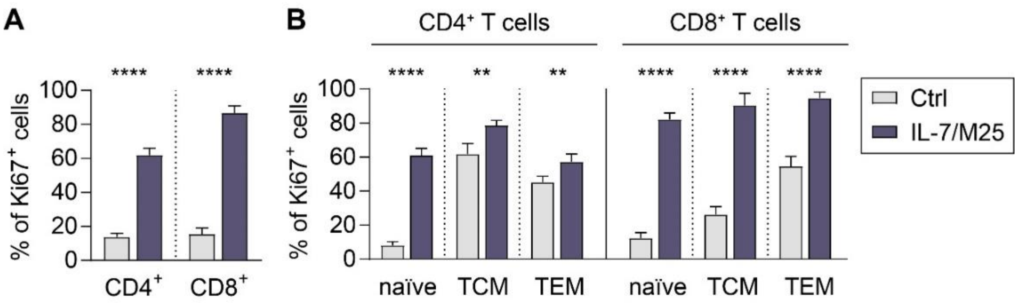

Figure S5. T cells are actively proliferating in scLN after IL-7/M25 treatment. Elevated IL-7 was induced by IL-7/M25 treatment three times every other day (n=4). Control animals (Ctrl) were treated with PBS (n=5). scLN were analyzed at day 7. Frequency of Ki67^{+} cells was determined by flow cytometry among **(A)** total CD4^{+} and CD8^{+} T cells and **(B)** the respective cell population with a naïve, TCM and TEM phenotype. Gray bars represent control mice, dark blue bars indicate IL-7/M25 complex treated mice. Shown is the mean of frequency ± SD. For statistical analysis, the student's t-test was applied. ** P≤ 0.01; **** P≤0.0001.

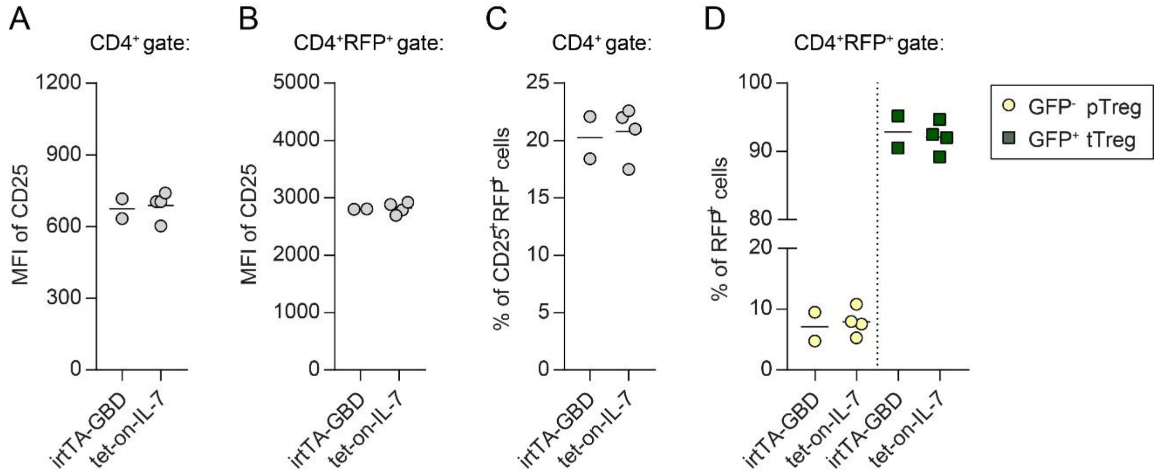

Figure S6. Separation of the sTG Ctrl group based on genotype. Siblings of dTG mice that express either the irtTA-GBD or the tet-on-IL-7 transgene were used as controls and shown in Figure 4 in one group. CD25 expression among **(A)** total CD4$^+$ T cells and **(B)** CD4$^+$-gated total Foxp3.RFP$^+$ Treg cells from Dex/Dox-treated Ctrl irtTA-GBD and Ctrl tet-on-IL-7 mice, as measured by the flow cytometric assessment of median fluorescent intensity (MFI). **(C)** Proportion of CD25$^+$Foxp3.RFP$^+$ among gated CD4$^+$ T cells. **(D)** Relative contribution of GFP$^-$ pTreg and GFP$^+$ tTreg cells among total CD4$^+$RFP$^+$ Treg cells.

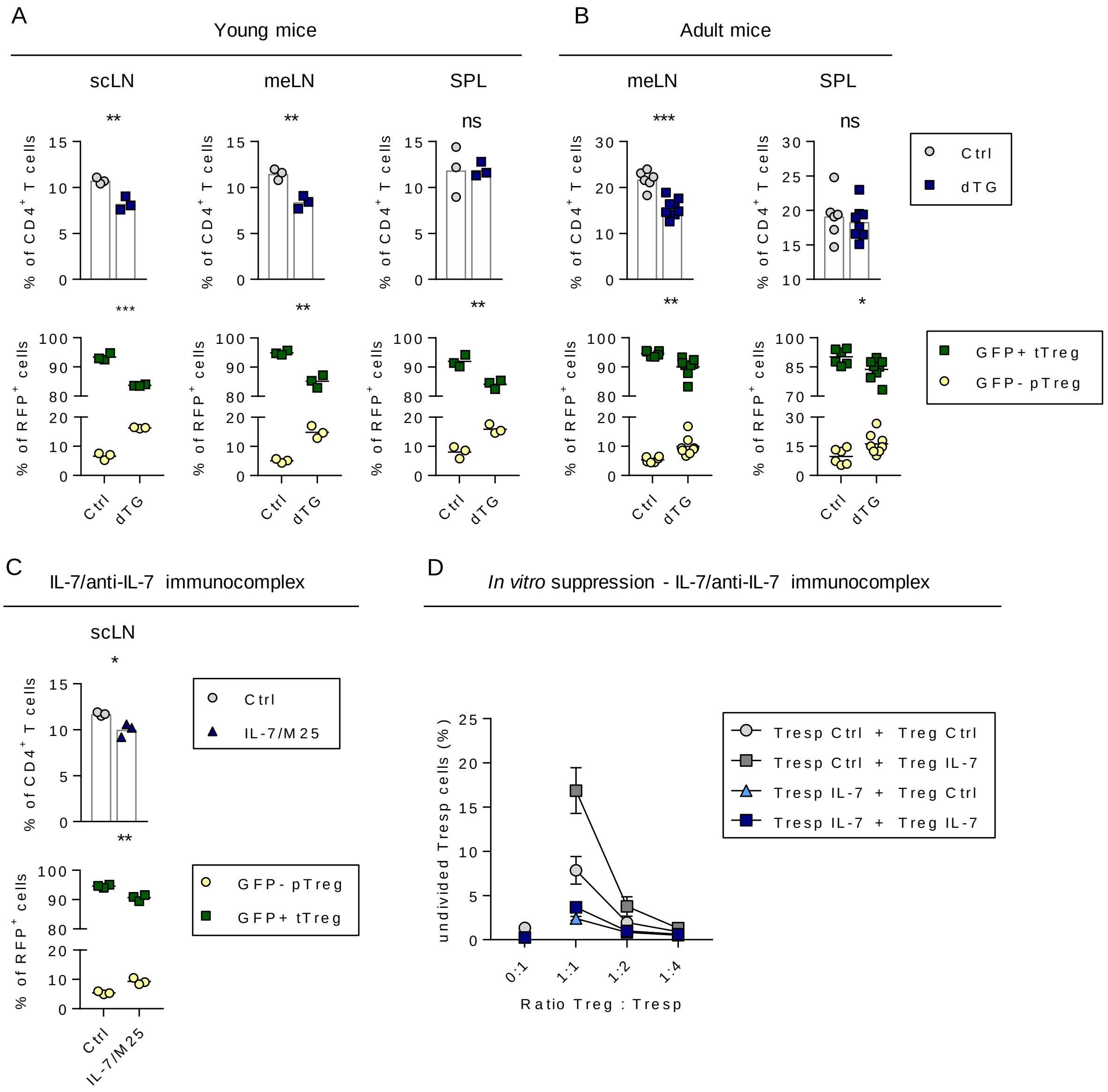

Figure S7. **Differential impact of increased IL-7 *in vivo* on developmental Foxp3$^+$ Treg cell sublineages and Foxp3$^+$ Treg cell-mediated suppression *in vitro*. (A, B)** IL-7 hyperexpression was induced in tet-on-IL7-irtTA-GBD × Foxp3$^{RFP/GFP}$ mice by 5-day Dex/Dox treatment. ScLNs, meLNs and SPL of young (4-week-old) and adult (16-week-old) mice were isolated on day 6 and analyzed by flow cytometry. Upper rows show the relative distribution of total RFP$^+$ Treg cells, while lower rows show the composite percentage of tTreg (green) and pTreg (yellow) subpopulations of Ctrl and dTG mice. **(C)** Foxp3$^{RFP/GFP}$ mice were treated with IL-7/M25 as described in the methods and scLNs were isolated at day 7. Symbols and horizontal lines indicate individual mice and mean values, respectively. For statistical analysis, the student's t-test was applied. n.s. P > 0.05; * P≤ 0.05; ** P≤ 0.01; *** P≤ 0.001. **(D)** *in vitro* suppression assay using Treg and Tresp cells isolated from IL-7/M25- or control-treated Foxp3$^{RFP/GFP}$ mice. Graph depicts the composite percentage of undivided Tresp cells under different culture conditions and Tresp:Treg cell ratios (triplicate wells per condition and sample),

as indicated. Symbols in the graph show mean and range of triplicate wells from one experiment. Ctrl – pool of 3 mice, IL-7/M25 – pool of 3 mice. For statistical analysis, two-way ANOVA with Bonferroni's multiple comparisons test was applied.

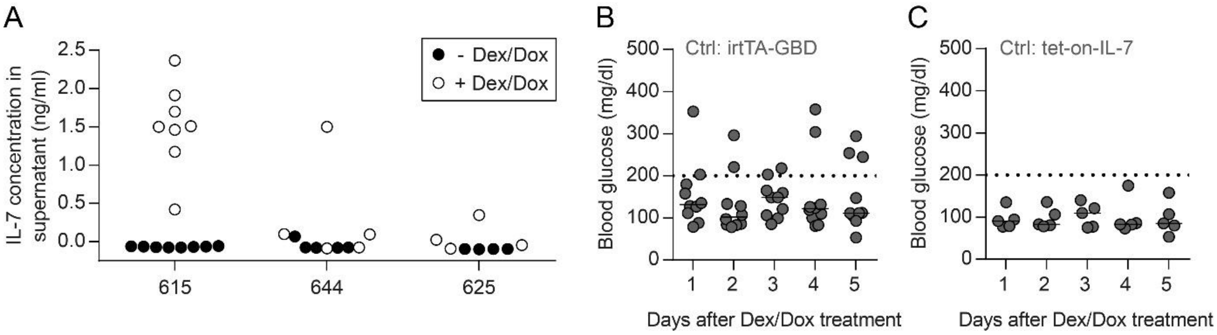

Figure S8. ***In vitro* IL-7 release in isolated islets. (A)** Isolated islets of founder 615 (n = 8), 644 (n = 5) and 625 (n = 4) were incubated in RPMI-1640 medium either in the absence (filled circles) or presence of Dex/Dox (open circles) for 48h. IL-7 was measured in the supernatant by ELISA. **(B, C)** Separation of the Ctrl group shown in Figure 6 based on genotype. Blood glucose concentrations after initiation of 5-day Dex/Dox treatment of BALB/c recipients transplanted with 600 allogenic islets from either **(B)** Ctrl irtTA-GBD (n = 10) or **(C)** Ctrl tet-on-IL-7 (n = 5) mice under the kidney capsule.
